# Risk factors associated with viral nervous necrosis in hybrid groupers in Malaysia and the high similarity of its causative agent nervous necrosis virus to reassortant red-spotted grouper nervous necrosis virus/striped jack nervous necrosis virus strains

**DOI:** 10.14202/vetworld.2019.1273-1284

**Published:** 2019-08-21

**Authors:** Nurshuhada Ariff, Azila Abdullah, Mohamed Noor Amal Azmai, Najiah Musa, Sandra Catherine Zainathan

**Affiliations:** 1 Department of Fisheries and Aquaculture, Faculty of Fisheries and Food Sciences, Universiti Malaysia Terengganu, Kuala Nerus, Terengganu, Malaysia; 2 Institute of Marine Biotechnology, Universiti Malaysia Terengganu, Kuala Nerus, Terengganu, Malaysia; 3 National Fish Health Research Division, Batu Maung, Penang, Malaysia; 4 Department of Biology, Faculty of Science, University Putra Malaysia, Serdang, Selangor, Malaysia; 5 Institute of Tropical Aquaculture and Fisheries Research, Universiti Malaysia Terengganu, Kuala Nerus, Terengganu, Malaysia

**Keywords:** epidemiology, quasispecies, reassortant, red-spotted grouper nervous necrosis virus-striped jack nervous necrosis virus, viral nervous necrosis

## Abstract

**Background and Aim:**

Viral nervous necrosis (VNN) is a serious disease of several marine fish species. VNN causes 100% mortality in the larval stages, while lower losses have been reported in juvenile and adult fish. This study aimed to detect the occurrence of VNN while identifying its associated risk factors and the genotypes of its causative agent in a hybrid grouper hatchery in Malaysia.

**Materials and Methods:**

A batch of newly hatched hybrid grouper fry (*Epinephelus fuscoguttatus* × *Epinephelus lanceolatus*) were followed from the larval stage to market size. Samples of the hybrid groupers, water, live feed, and artificial fish pellets were collected periodically from day 0 to 180 in the hybrid grouper hatchery. Reverse transcription-polymerase chain reaction (RT-PCR) and nested PCR amplifications were carried out on VNN-related sequences. The phylogenetic tree including the sampled causative agent of VNN was inferred from the coat protein genes from all known *Betanodavirus* species using Molecular Evolutionary Genetics Analysis (MEGA). Pearson’s correlation coefficient values were calculated to determine the strength of the correlation between the presence of VNN in hybrid grouper samples and its associated risk factors.

**Results:**

A total of 113 out of 146 pooled and individual samples, including hybrid grouper, water, and artificial fish pellet samples, demonstrated positive results in tests for the presence of VNN-associated viruses. The clinical signs of infection observed in the samples included darkened skin, deformation of the backbone, abdominal distension, skin lesions, and fin erosion. VNN was present throughout the life stages of the hybrid groupers, with the first detection occurring at day 10. VNN-associated risk factors included water temperature, dissolved oxygen content, salinity, ammonia level, fish size (adults more at risk than younger stages), and life stage (age). Detection of VNN-associated viruses in water samples demonstrated evidence of horizontal transmission of the disease. All the nucleotide sequences found in this study had high nucleotide identities of 88% to 100% to each other, striped jack nervous necrosis virus (SJNNV), and the reassortant strain red-spotted grouper NNV/SJNNV (RGNNV/SJNNV) isolate 430.2004 (GenBank accession number JN189932.1) (n=26). The phylogenetic analysis showed that quasispecies was present in each VNN-causing virus-positive sample, which differed based on the type of sample and life stage.

**Conclusion:**

This study was the first to confirm the existence of a reassortant strain (RGNNV/SJNNV) in hybrid groupers from Malaysia and Southeast Asia. However, the association between the mode of transmission and the risk factors of this virus needs to be investigated further to understand the evolution and potential new host species of the reassortant strain.

## Introduction

Viral nervous necrosis (VNN) is an acute and serious disease of fish caused by *Betanodavirus*, which was formerly known as viral encephalopathy and retinopathy, and has been reported in more than fifty fish species worldwide [1]. VNN in fish causes necrosis and vacuolation of the brain, spinal cord, and eyes [1], which leads to abnormal swimming behavior, and ultimately death. Massive disease outbreaks in Malaysia caused by VNN-associated viral infections have been reported in groupers (*Epinephelus fuscoguttatus*), Asian sea bass (*Lates calcarifer*), red snappers (*Lutjanus campechanus*), cobia (*Rachycentron canadum*), and golden pompano (*Trachinotus blochii*) [2-4]. Fishes in other Asian countries, such as Japan, Indonesia, Thailand, China, Singapore, and Taiwan, have also been affected by VNN [1]. Most of the reported cases of VNN occurred at the larval and juvenile stages [1].

Betanodaviruses, also known as nervous necrosis viruses (NNVs), are non-enveloped, are spherical, and are approximately 25-34 nm in diameter. The genome in these viruses consists of two molecules of positive-sense ssRNA: RNA1 (3.1 kb) encodes the replicase (110 kDa) and RNA2 (1.4 kb) encodes the coat protein (42 kDa). Complete nucleotide sequences of RNA1 and RNA2 have been reported for striped jack nervous necrosis virus (SJNNV) and other members of this group [1]. Previous studies have shown evidence of sequence divergence among the isolates of betanodaviruses from different geographical locations [1]. On the basis of phylogenetic analyses of the T4 variable region of their RNA2, betanodaviruses have been preliminarily clustered into four major genotypes, designated SJNNV, tiger puffer nervous necrosis virus (TPNNV), barfin flounder nervous necrosis virus (BFNNV), and red-spotted grouper nervous necrosis virus (RGNNV) [1]. In 2004, a fifth genotype group that originated from turbot, known as turbot nervous necrosis virus, was proposed by Johansen *et al*. [5]. These findings indicate the possibility of there being many other new strains based in other fish species that are still unknown.

The survivability of VNN under extreme environmental conditions has been reported previously. A previous study reported that the *Betanodavirus* genotypes are not strongly associated with specific host species, but tend to be correlated more with geographic location and water temperature [6]. A quantitative analysis conducted by Iwamoto *et al*. [7] using the cloned E-11 cell line showed distinct optimum growth temperatures for each of the four previously identified genotypes; specifically, RGNNV, SJNNV, TPNNV, and BFNNV displayed their highest virulence at 25-30°C, 20-25°C, 20°C, and 15-20°C, respectively. Other risk factors associated with VNN are fish age and fish size. VNN is frequently detected in small fish, although larger fish can be infected by its causative agent under certain circumstances, and this varies among fish species [8,9].

Genetic variation is generated by the accumulation of mutations during replication and their rearrangement by genetic recombination, as well as by genome segment reassortment in the case of segmented genomes [10]. RNA viruses, including betanodaviruses, have been shown to exhibit relatively high mutation rates, and RNA virus populations are extremely heterogeneous, which allows for greater adaptability and the rapid evolution of their RNA genomes [11]. RNA viruses demonstrate high mutation and self-copying error rates due to the lack of proofreading mechanisms during viral replication [11]. This genetic diversity allows a viral population to rapidly adapt to dynamic environments and evolve resistance to vaccines and antiviral drugs [12]. Genetic reassortment results in the production of quasispecies [13]. The quasispecies theory was initially formulated to understand and describe the evolutionary dynamics of RNA viruses. According to Lauring and Andino [10], a quasispecies is a cloud of diverse viral variants that are genetically linked through mutation, interact cooperatively on a functional level, and collectively contribute to the characteristics of the population. The quasispecies structure of NNVs was previously determined to investigate an outbreak of VNN at a barramundi (*L. calcarifer*) hatchery in Australia [14]. Variation in the viral capsid protein gene sequence from cell culture-derived viral populations and among individual fish suggested that each cohort of fish was infected with a different strain of virus (quasispecies). That molecular epidemiological study determined that the variation in the capsid genes of isolates obtained from this quasispecies was up to 3.3% compared to that of sequences sampled directly from fish tissues and 1.7% among individual fish within each cohort [14].

Reassortant viruses form as a result of the genetic shifts undergone by betanodaviruses due to the segmented nature of their genomes [15]. The VNN-causing viruses detected in gilthead sea bream (Sparus aurata) from the Mediterranean region were genetically characterized as belonging to reassortant strains, which were designated RGNNV/SJNNV [15]. To date, two types of reassortant strains have been described, RGNNV/SJNNV and SJNNV/RGNNV, whose genotype names refer to the donor genotypes of the polymerase/capsid protein genes (RNA1/RNA2, respectively) [15-18]. Similar to any other RNA viruses, Malaysian VNN-associated viral isolates could represent quasispecies, which allows them to adapt to their environment, replicate with a high mutation rate, and exhibit genetic diversity. It is vital to understand the reassortant strains of NNVs as they have the potential to produce new strains, which may have novel properties and enhanced pathogenicity [10].

This study aimed to detect and analyze VNN and isolates of its causative agent from hybrid groupers in Malaysia. This study described the first detection of a Malaysian NNV strain causing VNN that is highly similar to the reassortant strain RGNNV/SJNNV and may form quasispecies. This viral disease’s associated risk factors in Malaysian hybrid groupers were also assessed.

## Materials and Methods

### Ethical approval

No live fish were used during this study. All the samples and target organs examined were collected from the fish farm, and were then submitted to laboratory analyses for the detection of VNN and its associated virus.

### Experimental design and sample collection

A cross-sectional sampling was conducted at a hybrid grouper (*E. fuscoguttatus* × *Epinephelus lanceolatus*) hatchery in West Malaysia to detect the presence of VNN during a routine hybrid grouper fry production period from September 30, 2016, to March 29, 2017. The hatchery produced approximately two to three fish batches annually. The hybrid grouper fry was produced from a female parental tiger grouper (*E. fuscoguttatus*) broodstock from North Malaysia and a male parental giant grouper (*E. lanceolatus*) broodstock from West Malaysia. Information regarding the management of the hatchery was collected over a 6-month period of routine sampling on the farm. Basic data on the hatchery’s operating systems, such as the water system, hatchery plan, type of species culture, history of disease outbreaks, and husbandry practices, were obtained prior to sampling. Before this study, no attempts to detect VNN had been made on this farm. The movement of groupers from the nursery culture pond to grow-out tanks was followed to determine the prevalence of VNN and its associated risk factors in every life stage. Prior to sampling, epidemiological data, including the risk factors for and nature of disease outbreaks, were obtained at the farm. Risk factors associated with NNV infections and VNN disease, such as fish age, water system, artificial fish pellets, live feed, life stage (fry to adult), and water quality parameters, were evaluated during each sampling period. All the clinical signs associated with VNN were observed and noted during sampling. A total of 220 hybrid groupers’ fry (3000 larvae on day 0 in one pool; 85 larvae on day 10 in seventeen pools; 40 larvae on day 20 in two pools; 20 larvae on days 30, 40, 60, and 90 that were not pooled; and 14 larvae on day 180 that were not pooled) were freshly sampled and pooled accordingly throughout the study period (Table-1) [1].

**Table 1:** Number of samples processed and organs isolated based on the size of the fish.

Days post-hatching	Samples taken	No./vol. of samples	Pool	Size (OIE [1])	Isolated organ
0	Hybrid grouper larvae	3000	1	<2 mm	Whole body
	Water samples	500 mL	Duplicate		
10	Hybrid grouper larvae	85	5	<5 mm	Whole body
	Water samples	500 mL	Duplicate		
	Rotifers	500 mL			
20	Hybrid grouper larvae	40	2	~4-6 mm	Whole body
	Water samples	500 mL	Duplicate		
30	Hybrid grouper juveniles	20	No pool	~9-15 mm	Whole head
	Water samples	500 mL	Duplicate		
	Artificial fish pellets				
40	Hybrid grouper juveniles	20	No pool	~15-19 mm	Whole head
	Water samples	500 mL	Duplicate		
60	Hybrid grouper juveniles	20	No pool	~30-35 mm	Pool of brain and retina
	Water samples	500 mL	Duplicate		
	Copepods	500 mL			
90	Hybrid grouper juveniles	20	No pool	~40-45 mm	Pool of brain and retina
	Water samples	500 mL	Duplicate		
180	Hybrid grouper adult	14	No pool	~100 mm	Brain and retina
	Water samples	500 mL	Duplicate		
	Artificial fish pellets				

OIE=Office International des Epizooties

To confirm the horizontal transmission of the VNN-causing virus, 500-mL water samples were collected in duplicate from the ponds and tanks in which the hybrid groupers were reared on each day of sampling. To evaluate the risk factors associated with VNN, the water quality parameters were measured at a depth of approximately 1 m in the hybrid grouper rearing ponds and tanks during sampling. The water quality parameters measured included the temperature; dissolved oxygen (DO) content; pH; salinity; and concentrations of ammonia, nitrate, and nitrite, and all of these were measured *in situ* using handheld YSI™ meters. The recorded data were used to identify the possible association of the occurrence of VNN or its associated virus with the hatchery water quality at the different life stages of hybrid groupers. The samples of 500 mL of live feed, including copepods and rotifers, were collected on days 10 and 60, and the samples of artificial fish pellets were also similarly taken on days 60, 90, and 180 to identify the possible horizontal transmission of the VNN-causing virus among hybrid groupers. All the samples taken during the sampling periods were stored at −20°C prior to further analysis.

### Sample processing and RNA extraction

#### Fish sample processing

All the collected samples were processed and pooled accordingly depending on the fish size and sample type throughout the study period, as suggested by the Office International des Epizooties [1]. The hybrid grouper samples were aseptically dissected and processed according to the size of the fish (Table-1) [1]. The organ tissues were homogenized in a viral transport medium (VTM). The homogenized tissues were centrifugated at 8000× *g* for 5 min, and then the supernatant was collected for RNA extraction.

### Processing of water, live feed, and artificial formulated pellet samples

The samples of water (collected from day 0 to 180) and rotifers and copepods (collected on days 10 and 60) were centrifugated at 20,000× *g* for 20 min. The collected pellet was then re-suspended and homogenized in 500 μL of VTM. Artificial fish pellet samples (collected on days 30, 60, and 180) were homogenized using a tissue homogenizer and re-suspended in 500 μL of VTM. The homogenized samples (of water, rotifers and copepods, and artificial fish pellets) were then centrifugated again at 8000× *g* for 5 min. Then, 200 μL of the supernatant from each sample was collected and stored at −20°C prior to RNA extraction. The RNA extraction was conducted using the Nucleospin^®^ RNA Virus Kit (MACHEREY-NAGEL™, Bethelem, Pennsylvania, USA) according to the manufacturer’s protocols.

### Reverse transcription-polymerase chain reaction (RT-PCR) amplification

Throughout the study period, the extracted RNA samples were subjected to RT-PCR amplification to detect the presence of VNN-associated viruses (NNVs) using a MyTaq^™^ One-Step RT-PCR Kit (Bioline, Selangor, Malaysia) while following the manufacturer’s instructions. Single-step PCR was carried out to amplify the T1 region of the SJNNV RNA2 coat protein gene (Table-2) [1,19]. A PCR reaction mixture with a total volume of 50 µL was prepared containing 25 µL of 2× MyTaq One-Step Mix, 14.5 µL of diethylpyrocarbonate (DEPC)-treated water, 0.5 µL of reverse transcriptase, 1.0 µL of RiboSafe RNase inhibitor, 2 µL of the primer F1 (10 µM), and 2 µL of the primer R1 (10 µM), to which 5 µL of the extracted RNA was added. The amplification was done using a T100^™^ Thermal Cycler (Bio-Rad, Selangor, Malaysia), which was programmed to carry it out as follows: one cycle of RT at 45°C for 20 min and 95°C for 1 min, followed by 40 cycles of denaturation at 95°C for 10 s, annealing at 60°C for 10 s, and extension at 72°C for 30 s.

**Table 2:** Sets of primers used for the single-step PCR and nested PCR amplifications of the T1 and T4 regions, respectively, of the VNN-associated virus’s RNA2 genome.

Primer combination	5′-3′ sequence	Position	Product size	Reference
F1	5′-GGATTTGGACGTGCGACCAA-3’	143-162	1140 bp	[1]
R1	5′-GACAAGACTGGTGAAGCTGG-3′	1270-1289		
F2	5′-CGTGTCAGTCATGTGTCGCT-3′	592-611	426 bp	[1]
R3	5′-CGAGTCAACACGGGTGAAGA-3′	1017-998		
RGNNV-NFRG	5′-CAGCGAAACCAGCCTGCAGG-3′		258 bp	[19]
RGNNV-NRRG	5′-ACCTGAGGAGACTACCGCTG-3′			

PCR=Polymerase chain reaction, VNN=Viral nervous necrosis, RGNNV=Red-spotted grouper nervous necrosis virus

A nested PCR was carried out according to de la Peña *et al*. [19] using the F2-R3 primer set targeting the T4 region gene sequence of the SJNNV coat protein (Table-2) [1,19]. For the primary PCR reaction, one step of RT-PCR amplification was conducted using the primer set F2 and R3, which amplifies amplicons with an expected size of 426 bp. A PCR reaction mixture with a total volume of 25 µL was prepared, containing 12.5 µL of 2× MyTaq One-Step Mix (Bioline), 6.75 µL of DEPC-treated water, 0.25 µL of reverse transcriptase, 0.5 µL of RiboSafe RNase inhibitor, 1 µL of F2 (10 µM), and 1 µL of R3 (10 µM), to which 3 µL of the extracted RNA was added. For the primary reaction, the amplification was programmed to be carried out as follows: one cycle of RT at 45°C for 20 min and polymerase activation at 95°C for 1 min, followed by 40 cycles of denaturation at 95°C for 10 s, annealing at 60°C for 10 s, and extension at 72°C for 30 s. The PCR products were then subjected to a secondary PCR reaction to amplify products with a size of 258 bp using the primer set RGNNV-NFRG and RGNNV-NRRG. The secondary PCR reaction was carried out with 12.5 µL of 2× My Taq^™^ Mix (Bioline), 8.5 µL of DEPC-treated water, 1 µL of RGNNV-NFRG (10 µM), and 1 µL of RGNNV-NRRG (10 µM), to which 2 µL of the PCR product from the primary PCR was added. The amplification was conducted under the following conditions: one cycle at 95ºC for 1 min, followed by 35 cycles of denaturation at 95°C for 15 s, annealing at 58°C for 15 s, and extension at 72°C for 10 s.

The amplified PCR products from both reactions were then analyzed by electrophoresis, which was run for 45 min at 70 V on 1.7% (w/v) agarose gel in tris-acetate-ethylenediaminetetraacetic acid buffer and stained with SYBR^®^ Safe – DNA Gel Stain (Invitrogen, Waltham, Massachusetts, USA). A synthetic positive control based on the sequence of SJNNV (GenBank accession number D30814.1) was used in this study. The expected bands were excised and sent for sequencing analysis to First Base Laboratories Sdn Bhd (Selangor, Malaysia). The DNA sequencing results were then used for phylogenetic analysis. The sequences were used to query the NCBI BLAST database to confirm their likely identity. The multiple alignments obtained were then aligned using Clustal X2.0.12 with other *Betanodavirus*-related sequences. Finally, a phylogenetic analysis was performed based on the coat protein genes of the samples and the highly similar published sequences of other viruses in Molecular Evolutionary Genetics Analysis software (MEGA 7.0.26) (Pennsylvania State University, USA). The phylogenetic tree was constructed using the neighbor-joining method. Evolutionary distances were computed using the maximum composite likelihood method.

### Statistical analysis

Epidemiological data, including fish age, fish size, and water quality parameter values, were analyzed by computing Pearson’s correlation coefficient (r) values (in SPSS statistics software version 20, IBM, USA) to determine the strength of their correlations with the presence of VNN/NNV in the hybrid grouper samples. For this analysis, the r values could range between −1 and 1, where positive r values indicated that there was a positive linear correlation, and negative r values meant that there was a negative linear correlation. To interpret the strength of the relationships between variables, r values from 0.00 to 0.39 were considered as representing weak correlations, those from 0.40 to 0.59 meant that the variables were moderately correlated, and those from 0.60 to 1.0 represented strong correlations. For the correlation to be concluded statistically significant, its associated p-value had been <0.05.

## Results

### Hatchery management

Throughout the study period, proper husbandry practices, good hygiene, or quarantine were never enforced in the hatchery (Table-3). The maintenance of biosecurity between different parts of the facility was also neglected. The hatchery pumped water directly into rearing containers from the nearby estuary, with no ozonation system or proper filter used to filter the incoming and outgoing water. The pre-hatch larvae obtained at day 0 had been cultured in circular spawning tanks in seawater (temperature = 32.3°C, salinity = 25.71 ppt). Then, at day 2, the larvae were transferred to earthen ponds (temperature = 32.3°C, salinity = 25.71 ppt) with green water and reared until day 25. During this period, the larvae were fed with rotifers and copepods produced in-house 6-8 times a day depending on the amount of rotifers given. The earthen ponds were covered and surrounded with black shading nets to prevent the exposure of the larvae to direct sunlight. Once the larvae reached an age of 26 days, they were transferred to and divided among six circular polyvinyl chloride tanks (water temperature = 32°C, salinity = 26 ppt) and reared until they reached an age of 180 days (the grow-out/adult stage). At 28 days, the larvae were fed with artificial fish pellets, and no trash fish was given to them as food.

**Table 3:** Hatchery management with respect to the husbandry practices in the fish farm.

Factors	Description	
Farmers’ attire and equipment	Similar attire and equipment used among different parts of the hatchery The utensils or tools used to feed the fish are not changed regularly	
Disinfection	No disinfection techniques practiced by the farmers prior to feeding, changing water, and tank cleaning	
Stocking density	Stocking density of hybrid groupers	

	**Fish size (cm)**	**Stocking** **density**

	2.0-2.54	4000-6000
	3.81	3000
	5.08	1500
	6.35	1000

Rearing conditions	The groupers were reared at temperatures ranging between 29.3 and 32.5°C and salinities between 18.5 and 26.8 ppt	
Water supply	Inadequate filtration (only sand and sediment filter) and absence of UV sterilization of the incoming water supply to the hatchery	
Egg ozonation	No ozonation/disinfection of hatching eggs	
Type of species cultured	Multiple species were reared in the same culture area, such as snapper and sea bass	
Feeding rate	No specific amount or frequency of feeding; the groupers were fed frequently each day depending, on their size	
Fish shelter	A fish “house” or “cave,” which was made of a pipe or net, was provided as an area for fish to hide/shelter from larger fish	
Change of water	Larval stages: Bottom cleaning twice a day (siphoning out the residual food), with the addition of new water Juvenile stages: Water changed twice a day	

UV=Ultraviolet

### Water quality parameters

The water quality parameters measured on each day of sampling from day 0 to 180 demonstrated values within their normal range in a hatchery setting (Table-4). The water temperature was lower (29°C) from day 30 to 60 compared to that during the rest of the study period. Similarly, the water salinity was observed to be lower between days 30 and 40 (18-19 ppt). Significantly, the DO in the culture water was higher from day 30 to 180 (5.52-5.74 mg/L) compared with that from day 0 to 20 (4.42-4.52 mg/L). The water pH reached its lowest value at day 180, and was the highest at day 30, but was never lower than 7.

**Table 4:** Water quality parameters measured during sampling throughout the study.

Parameter	Day							

	0	10	20	30	40	60	90	180
Temperature, °C	32.3	32.5	32.4	29.3	29.8	29.5	31.2	31.6
Pressure, mmHg	754.6	754	754	755	755	755	755	759.2
DO, mg/L	4.42	4.5	4.55	5.52	5.4	5.45	5.56	5.74
SPC, μs/cm	40544	50632	50632	50630	50630	50630	50630	32384
TDS, mg/L	26351	32890	32890	32890	32890	32890	32890	21047.6
Salinity, ppt	25.71	24.75	26.8	19.5	18.5	21.4	19.35	20.2
pH	7.45	7.2	7.35	7.6	7.5	7.55	7.4	7

DO=Dissolved oxygen, SPC=Specific conductance, TDS=Total dissolved solids

### Clinical signs

**Figure-1:**
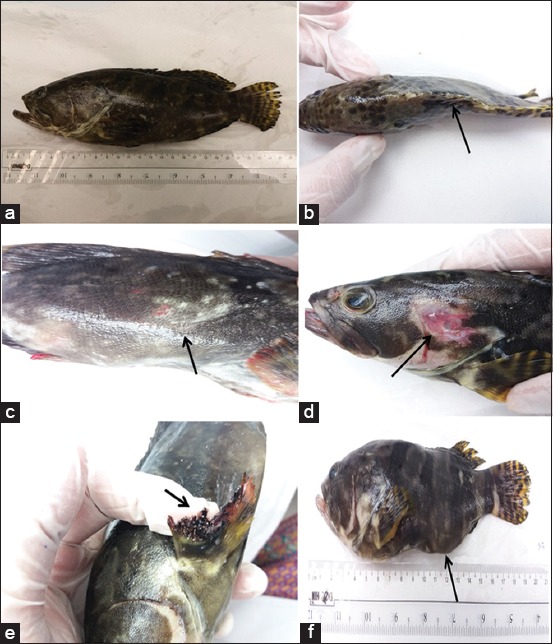
Clinical signs of viral nervous necrosis observed in grouper samples at day 180: (a) Darkened skin, (b) backbone deformation, (c) body lesions, (d) fin lesions, (e) rotten fins, and (f) abdominal distension.

Most of the grouper samples collected from day 0 to 90 did not demonstrate any clinical symptoms of VNN, although such signs did manifest at day 180. The grouper samples collected at this point showed clinical signs such as darkened skin, backbone deviation, lesions on the body and fins, rotten fins, and distended abdomens (Figure-1).

### Single-step PCR analysis

The single-step PCR analysis did not demonstrate positive results for the presence of VNN-associated virus in any samples, including in grouper, water, artificial fish pellet, and live feed (rotifers and copepods) samples.

### Nested PCR analysis

In the nested PCR analysis, most of the fish samples collected on each sampling day (day 10-180) demonstrated positive results for the presence of VNN-associated virus, except on day 0 (Table-5) [19]. The water samples were positive for the presence of VNN-causing virus except at days 60 and 90, whereas the live feed (rotifers and copepods) collected at days 60 and 90 were negative for the presence of VNN-causing virus. The artificial fish pellets demonstrated positive results for VNN-associated viral presence at day 30 and negative results at days 60 and 180. Reproductive fluids (eggs and sperm) collected at the initial stage of the study were negative for the VNN-causing virus (data not shown). However, because samples of reproductive fluids for this batch of samples were unavailable, the possibility of vertical transmission of the disease-causing NNV could not be ruled out. The frequency of occurrence of the VNN-causing virus in grouper samples was high from day 10 to 60, which meant that the incidence of VNN in groupers was higher in the larval stage.

**Table 5:** The total number of VNN-causing virus-positive samples detected each day, based on the nested PCR analysis using the RGNNV primer set of de la Peña *et al*. [19].

Day	Sample type	Number of samples	Pool	Result (VNN)	Occurrence of VNN (%)
0	Post-hatch groupers’ larvae	3000	1	-	0
	Water samples			+	
10	Grouper larvae	85	5	15/17+	88
	Water samples			+	
	Rotifers			-	
20	Grouper larvae	40	2	2/2+	100
	Water samples			+	
30	Grouper larvae	20	No pool	13/20+	65
	Water samples			+	
	Artificial fish pellets			-	
40	Grouper juveniles	20	No pool	17/20+	85
	Water samples			+	
60	Grouper juveniles	20	No pool	20/20+	100
	Water samples			-	
	Artificial fish pellets			+	
	Copepods			-	
90	Grouper juvenile	20	No pool	11/20+	55
	Water sample			-	
180	Grouper				
	Eyes	14	No pool	5/14+	
	Brains	14	No pool	12/14+	86*
	Water samples			+	
	Artificial fish pellets			+	

* The NNV-positive brain samples were treated as representing the whole occurrence of the VNN virus in both eye and brain samples. PCR=Polymerase chain reaction, VNN=Viral nervous necrosis, RGNNV=Red-spotted grouper nervous necrosis virus, NNV=Nervous necrosis virus

### Epidemiological data analysis

Analysis of Pearson’s correlation coefficient values was done to establish whether there was a relationship between the presence of VNN/NVV in hybrid groupers and different water quality parameters, fish size, and fish age. The significant risk factors associated with VNN viral infection identified in this study included the temperature, DO, salinity, and ammonia concentration in the rearing water, as well as fish size and life stage (p<0.01) (Table-6). Negative relationships were demonstrated between both temperature and salinity and VNN-causing virus presence, which indicated that grouper samples were less susceptible to VNN at higher temperatures (r=−4.06) and higher salinity (r=−6.09). Strong and significant positive relationships were demonstrated between the presence of the VNN-causing virus in groupers and the DO in water samples, fish size, and life stage (i.e. infection rates were higher in adult than larval groupers). Although the ammonia concentration in the water did not influence the presence of VNN in grouper samples strongly (r=0.19), it was still concluded to be a significant risk factor. There was a statistically significant (p<0.01) relationship between all the water quality parameters examined, except for pH (p>0.05), with the presence of VNN-causing virus in groupers. Adult groupers were found to be more susceptible to VNN compared to other life stages (p<0.01).

**Table 6:** Results of assessments of the strength of the relationship between the presence of VNN or its causative agent in groupers or water samples and fish size, fish age, and water quality parameters based on the analysis of Pearson’s correlation coefficients.

Risk factors	p-value	r-value
High temperature: Lower rate of VNN infection in grouper samples	<0.01	−4.06
High concentration of DO		
Higher rate of VNN infection in grouper samples	<0.01	0.88
Higher rates of VNN infection in water samples	<0.01	0.924
High salinity		
Grouper samples were less susceptible to VNN infection	<0.01	−6.79
Water samples were more susceptible to VNN infection	<0.05	0.403
Concentration of ammonia: Positively influenced the presence of VNN in groupers	<0.05	0.19
Larger fish size		
Longer fish were more susceptible to VNN infection	<0.01	0.842
Heavier fish were more susceptible to VNN infection	<0.01	0.701
Life stage/age: Higher rates of VNN infection were recorded in adults compared to younger (larval and juvenile) groupers	<0.01	0.882

VNN=Viral nervous necrosis, DO=Dissolved oxygen

### Sequence and phylogenetic analyses

**Figure-2:**
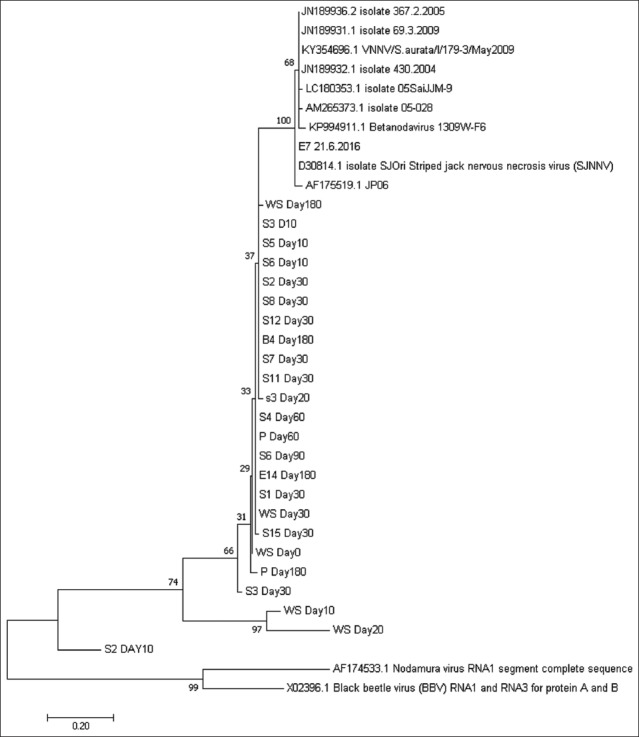
Unrooted phylogenetic tree constructed for 426 bp segments within the T4 region of RNA2 comparing the nucleotide sequences obtained in this study to the coat protein sequences of other betanodaviruses. D30814.1=coat protein gene of striped jack nervous necrosis virus (SJNNV) (RNA2), JN189932.1=red-spotted grouper nervous necrosis virus (RGNNV)/SJNNV from *Solea senegalensis*, KY354696.1=RGNNV/SJNNV from *Sparus aurata*, JN189931.1=RGNNV/SJNNV opistobranchia betanodavirus, AM265373.1=Iberian isolate, KP994911.1=Chinese isolate, LC180353.1=SJNNV Japanese isolate, AF175519.1=*Dicentrarchus labrax* SJNNV, AF174533.1=*Nodamura virus*, and XO2396.2=*Black beetle virus*.

All 25 NNV-positive samples collected from day 0 to 180 were sent for sequencing analysis, which was done for 257 bp of the T4 region of the RNA2 of SJNNV, and included an isolate obtained from preliminary work (E7 preliminary). From the sequencing results, a phylogenetic analysis was then conducted to confirm the likely similarities and quasispecies nature of the VNN-associated viral isolates collected within the samples in this study. Multiple alignments of the nucleotide sequences found in this study revealed that the entirety of the samples collected from day 0 to 180 (except at day 40) contained the same member of the *Betanodavirus* genus, and also demonstrated high similarity to SJNNV and RGNNV/SJNNV. Although the samples from day 40 were positive for the VNN virus based on PCR analyses, no similarities were found between them and other known NNV isolates based on the sequencing analysis. This could be due to there having been insufficient PCR product for sequencing analysis. All the samples demonstrated 94-99% nucleotide sequence identity to RGNNV/SJNNV reassortant strains, including JN189932.1, KY354696.1, and JN189931.1. The NNV-positive samples showed similarities ranging from 88 to 100% to the coat protein gene (RNA2) of SJNNV (D30814.1), JN189932.1 (RGNNV/SJNNV from *Solea senegalensis*), KY354696.1 (RGNNV/SJNNV from *S. aurata*), JN189931.1 (RGNNV/SJNNV; Opisthobranchia *Betanodavirus*), AM265373.1 (Iberian isolate), KP994911.1 (Chinese isolate), LC180353.1 (SJNNV Japanese isolate), and AF175519.1 (*Dicentrarchus labrax* SJNNV). The following two outgroups were included in the analyses: AF174533.1 (*Nodamura virus*) and XO2396.1 (*Black beetle virus*). The phylogenetic analysis suggested that the isolated samples were grouped within the RGNNV/SJNNV reassortant strain (Figure-2). The nucleotide sequences obtained in this study also revealed the quasispecies nature of the sequences from each of the different types of samples (grouper, water, and pellet samples), as well as among the different life stages (Table-7).

**Table 7:** Percent similarities of the nucleotide sequences of the capsid protein gene (RNA2) determined to characterize the quasispecies nature of the nucleotide sequences found in this study and published sequences retrieved from GenBank.

Accession No.	1	2	3	4	5	6	7	8	9	10	11	12	13	14	15	16	17	18	19	20	21	22	23	24	25
D30814.1	99	99	97	97	97	98	97	96	94	100	99	99	99	100	100	97	98	100	97	97	99	98	99	96	98
JN189932.1	99	99	96	96	96	97	96	95	94	99	98	99	99	99	97	96	97	99	96	96	98	97	98	95	97
KY354696.1	99	99	97	96	97	98	97	96	94	98	98	99	99	96	99	97	97	98	97	96	98	98	98	96	98
JN189931.1	99	99	97	96	97	98	97	96	94	98	98	99	99	96	99	97	97	98	97	96	98	98	98	96	98
AM265373.1	99	98	96	96	96	97	96	95	93	97	98	98	98	96	99	96	96	97	96	96	98	97	98	95	97
KP994911.1	97	98	97	97	97	95	95	95	92	98	97	98	98	98	98	95	96	98	96	97	97	98	97	96	96
LC180353.1	97	98	97	97	97	96	96	96	93	99	97	98	98	99	99	96	96	99	98	97	98	96	98	96	97
AF175519.1	98	98	98	95	95	97	96	94	93	99	98	98	98	99	99	96	96	99	96	95	98	97	98	95	97

*Data are presented as percentage similarities of 426 bp gene segments among sequences. Sequence variation was observed among the samples found in this study as well. 1=E7/21.6.2016 (preliminary sample), 2=ws/day0/30.9.2016, 3=S2/day10/10.10.2016, 4=S3/day10/10.10.2016, 5=S5/day10/10.10.2016, 6=S6/day10/10.10.2016, 7=ws/day10/10.10.2016, 8=S3/day20/20.10.2016, 9=*ws/day20/20.10.2016, 10=S1/day30/30.10.2016, 11=S2/day30/30.10.2016, 12=S3/day30/30.10.2016, 13=S7/day30/30.10.2016, 14=S8/day30/30.10.2016, 15=S11/day30/30.10.2016, 16=S12/day30/30.10.2016, 17=S15/day30/30.10.2016, 18=*ws/day30/30.10.2016, 19=S4/day60/29.11.2016, 20=*p/day60/29.11.2016, 21=S6/day90/29.12.2016, 22=brain4/day180/29.3.2017, 23=eye14/day180/29.3.2017, 24=*ws/day180/29.3.2017, and 25=*p/day180/29.3.2017. *p indicates an artificial fish pellet sample, *ws indicates a water sample. D30814.1=coat protein gene of SJNNV (RNA2), JN189932.1=RGNNV/SJNNV from *Solea senegalensis*, KY354696.1=RGNNV/SJNNV from *Sparus aurata*, JN189931.1=RGNNV/SJNNV opistobranchia betanodavirus, AM265373.1=Iberian isolate, KP994911.1=Chinese isolate, LC180353.1=SJNNV Japanese isolate, and AF175519.1=*Dicentrarchus labrax* SJNNV. RGNNV=Red-spotted grouper nervous necrosis virus, SJNNV=Striped jack nervous necrosis virus

## Discussion

A total of 113 out of 146 pooled and individual samples demonstrated positive results for the presence of the viral causative agent of VNN, including hybrid grouper, water, and artificial fish pellet samples. The first detection of VNN occurred at day 10 post-hatch (in larvae), and then VNN continued to be detected until day 180 (in adults). The detection of the VNN-causing virus in water samples provided evidence of horizontal transmission. The VNN-associated risk factors identified in this study included water quality parameters and fish age, size, and life stage. All the nucleotide sequences obtained in this study had a high nucleotide identity of 88-100% with each other, SJNNV, and the reassortant strain RGNNV/SJNNV isolate 430.2004 (GenBank accession number JN189932.1). The phylogenetic analysis done showed that quasispecies was present in each NNV-positive sample, depending on the type of sample and grouper life stage.

Several clinical symptoms related to VNN were observed at day 180, including darkened skin pigmentation, backbone deviation, abdominal distention, skin lesions, and fin erosion. Backbone deviation has been shown to occur due to the occurrence of vacuolation in the central nervous system, especially the spinal cord [1,20]. Similarly, in other fish with VNN, skin color variations, anorexia, lethargy, or swim bladder hyperinflation are also frequently observed [1,15,21]. In a previous study of VNN in another fish species, this disease caused fish fingerlings to exhibit abnormal swimming behavior, darkened skin coloration, and loss of appetite prior to mass mortality [2,5]. Our findings also indicated that the larval and juvenile groupers in this study (<180 days of age) had subclinical infections, whereas the studied adult groupers exhibited clinical infections.

The NNV-positive water samples found throughout the rearing of different life stages indicated that horizontal transmission was possible via the rearing tank water. The horizontal transmission of the virus-causing VNN has been known to occur via infected fish, contaminated trash fish, contaminated feed pellets, and contaminated water supplies [1,22,23]. A previous traditional epidemiological study indicated that the horizontal transmission of VNN occurred between two cohorts of fish, which was due to a VNN outbreak at a barramundi hatchery in Australia [24]. The detection of the VNN-causing virus in artificial fish pellets at days 60 and 180 in the present study indicated that contamination occurred due to handling and the lack of biosecurity measures. A previous study proposed that horizontal transmission can occur via virus-contaminated food based on the detection of *Nodavirus* in live bait obtained from the infected farms [1]. Horizontal transmission also possibly happens due to inadequate biosecurity measures or the presence of asymptomatic NNV carriers [24], and this was evident in the present study’s findings as the fish farm that supplied our samples lacked biosecurity measures. Live feed (rotifers and copepods) sampled at days 10 and 60 was negative for the presence of NNVs. Similarly, live feed samples (rotifers and *Artemia*) collected from an Australian barramundi hatchery were negative for the presence of VNN-causing virus [14]. In this study, we were unable to demonstrate the vertical transmission of VNN due to the unavailability of reproductive samples from the broodstock. The possibility of vertical transmission is still ambiguous, as the first detection of VNN was made at day 10, and the onset of VNN was consistent throughout the life stages examined.

The VNN-associated risk factors identified in this study included the temperature, DO, salinity, ammonia concentration in the water, fish size (adult > larvae), and life stage (age). Similarly, infection with the VNN virus has previously been shown to be influenced by host factors, such as age and environmental factors, particularly temperature [25]. Previous studies have shown that VNN first occurs from as early as 10 days post-hatching (dph) to 50 dph, and in some cases, fish beyond 50 dph in age can be affected, particularly strongly by the viral disease during periods of stress [26,27]. An association between VNN outbreaks and husbandry-related stress factors, such as suboptimal feed, suboptimal water quality, crowding, transport, and repeated spawning of broodstock, has been reported in several studies [5]. In this study, salinity was shown to be more negatively correlated with the presence of VNN-causing virus in hybrid grouper samples than in water samples (with a positive and moderate correlation). Salinity does not seem to have had any influence on the occurrence of VNN disease in previous studies, as outbreaks have also been reported in freshwater species [1]. Similarly, a lower rate of occurrence of VNN was found in grouper samples collected at higher temperatures (with there being a negative and moderate correlation between temperature and VNN incidence). The water temperature throughout the rearing of the life stages of the groupers in this study was between 29 and 33°C, which is higher than the optimum growth temperature for RGNNV *in vitro* (25-30°C). The temperature required for the RGNNV strain to induce VNN disease varies among fish species; for example, it is from 23 to 25°C for sea bass, but from 28 to 30°C for different species of groupers [28]. Water temperatures around 16-28°C were revealed to influence the development of VNN disease in RGNNV-positive seven-band grouper [1]. In the humpback grouper (*Cromileptes altivelis*), water temperatures higher than 31°C inhibited the spread of RGNNV, despite the fact that more mortalities and the earlier onset of the disease were observed at higher temperatures (35°C) [25]. The association between high water temperatures and the presence of diseases in older fish has previously been reported in groupers and sea bass. However, all the NNV-positive samples found in this study showed high similarities with the reassortant strain, which was thought to have a difference in optimum growth temperature from that of other genotypes. Previously, VNN was observed to occur in Senegalese sole regardless of rearing temperature during experimental challenges with a reassortant strain (RGNNV/SJNNV) [29]. Notably, a study by Panzarin *et al*. [27] showed that sudden increases in the viral titer of the RGNNV and RGNNV/SJNNV strains were observed at 30°C. The reassortant strain (RGNNV/SJNNV), with its RNA1 of the RGNNV-type, was able to replicate effectively at a high temperature (30°C) [27]. This finding was consistent with the present study, wherein the range of water temperatures at which fish were cultured was between 29 and 33°C.

Larger and adult fish seemed to be more frequently affected by VNN than smaller and younger (larval and juvenile) fish, respectively, based on the present study. Age is definitely a major risk factor for susceptibility to VNN disease, as was previously found in a barramundi hatchery [24] and other experiments. The infection observed in barramundi was subclinical, and the fish were raised to an age of up to 64 days despite there being a 93.6% infection frequency [24]. Based on the data from this study, groupers more than 10 days old are susceptible to VNN disease. No previous studies have reported that high DO and ammonia content were risk factors for VNN. In general, a high DO, as well as ammonia and nitrite toxicity, can affect fish respiration [30]. When water is supersaturated with oxygen (hyperoxia), the swim bladder becomes overinflated, and this leads to buoyancy problems, especially in small fishes [30]. Hatchery management and biosecurity measures were other major risk factors in this study, due to the absence of the proper implementation of biosecurity measures in the hatchery. The VNN outbreaks previously observed in a barramundi hatchery in Australia were initiated by flaws in the water supply system and spread due to the failure of biosecurity measures [24].

The phylogenetic analysis demonstrated that the VNN-associated viral sequences detected in this study could be classified as RGNNV/SJNNV reassortant strains (n=26), and that they represent quasispecies. These results revealed that different quasispecies affected the samples across different life stages. This was the first study that has detected NNV strains highly similar to RGNNV/SJNNV in Malaysian groupers, and in South-East Asia as a whole, as no work has been conducted on the molecular epidemiology of VNN in these regions previously. The RGNNV genotype has been found to cause diseases in many warm-water fish species, including groupers [23]. RGNNV/SJNNV reassortant strains were detected by Panzarin *et al*. [18] in their phylogenetic analysis, which revealed the presence of RGNNV/SJNNV (in 23/120 samples) reassortant strains in South Europe. The reassortment of *Betanodavirus* isolates occurs when the virus comes to possess the RNA2-RGNNV genotype and the RNA1-SJNNV genotype simultaneously (RGNNV/SJNNV) [18,21]. RGNNV/SJNNV reassortant strains have previously affected the European sea bass, gilt-head seabream (*S. aurata*), and Senegalese sole (*S. senegalensis*) in Portugal and Spain [17,18].

Similar to the findings of the present study, RGNNV/SJNNV reassortant strains have previously been found to infect the same fish. Reassortment events occur in RNA segmented viruses, enabling the virus to undergo faster evolution and giving it the ability to infect new hosts and avoid the host’s immune system [21,31,32]. The RNA2 genome of the reassortant RGNNV/SJNNV is different from the RNA2 genome of SJNNV [21]. SJNNV alone is incapable of causing disease in European sea bass, but the RGNNV/SJNNV reassortant strain can cause clinical signs and mortality to manifest in this same species, as the reassortant strain is more virulent. The combination of segments comprised of both SJNNV and RGNNV is successful, and able to cause disease in the target host [16]. Similarly, the analysis of the genomes of betanodaviruses from cultured marine fish species, such as Asian sea bass (*L. calcarifer*), golden pompano (*T. blochii*), and brown-marbled grouper (*E. fuscoguttatus*), in Malaysia revealed that they belonged to the RGNNV genotype group. However, the VNN-associated strains detected in this study were revealed to possess RGNNV/SJNNV genotypes. New strains such as these possibly come into existence to enable the virus to undergo faster evolution and have higher virulence. According to Costa and Thompson [21], almost all RGNNV/SJNNV chimeras are the result of a single reassortment event that occurred in South Europe during the early 1980s. A Malaysian *Betanodavirus* isolate was previously found to be composed of an older RNA2 gene and a younger RNA1 gene [31]. However, to confirm the genetic reassortment in the samples examined, complete identification of the genetic data from both genomic components (RNA1 and RNA2) is required [16,18,32]. The emergence of VNN-causing strains that are highly similar to RGNNV/SJNNV reassortant strains in Malaysian groupers indicates that the VNN-causing virus is evolving, mutating, and adapting to the environment existing in hatcheries to become more virulent.

## Conclusion

This was the first epidemiological study of VNN in Malaysian groupers, and was supported by the identification of disease-associated risk factors and was able to detect RGNNV/SJNNV reassortant strains. It is important to note that the virulence of *Betanodavirus* strains is highly dependent on environmental factors, and in this study, such important factors were identified that included water quality parameters and host factors, including fish age and life stage. To date, many studies have highlighted the risk factors associated with the development of this viral disease. However, a limited number of studies have demonstrated the epidemiology of VNN. Thus, the findings of this study may possibly help improve our understanding of the epidemiology and risk factors of VNN in Malaysia. This will enable fish farmers to improve biosecurity measures to reduce the impacts of this viral disease on cultured fish. The observed emergence of RGNNV/SJNNV reassortant strains resulting in quasispecies in each sample should serve as an early warning to the grouper industry in Malaysia. The importance of good biosecurity measures should be further emphasized in the grouper industry, including the implementation of at least cost-effective and low-level biosecurity measures, the use of water filters at the water inlet, proper water filtration systems, restrictions to human movements within hatcheries, and the implementation of disinfection protocols.

## Authors’ Contributions

SCZ supervised the research as the main researcher. AA, MNAA, and NM co-supervised NA’s laboratory work. NA carried out all the laboratory work and data analyses. NA carried out the statistical analyses with the help of SCZ and MNAA. NA wrote the manuscript with the help of SCZ, AA, MNAA, and NM. All authors read and approved the final manuscript.
